# Monitoring neonatal brain function: recent advances in functional near-infrared spectroscopy (fNIRS)

**DOI:** 10.3389/fped.2025.1689905

**Published:** 2026-01-13

**Authors:** Fei Xu, Jie Li, Chao Zheng, Lanlan Mi

**Affiliations:** 1 Department of Neonatology, Shangrao Municipal Hospital, Shangrao, Jiangxi, China; 2 Department of Pharmacy and Medical Devices, The Third People's Hospital of Shangrao City, Shangrao, Jiangxi, China; 3 Department of Neonatology, Shanghai Children’s Medical Center, School of Medicine, Shanghai Jiao Tong University, Shanghai, China

**Keywords:** brain network imaging, fNIRS, neonate, neurodevelopmental outcomes, neuroscience, review

## Abstract

Understanding functional brain development in neonates represents a critical frontier in neuroscience. Due to the high plasticity of the infant brain, early detection of functional abnormalities and timely interventions are essential to improving long-term neurodevelopmental outcomes. However, progress in this field has been limited by the constraints of conventional neuroimaging modalities. The emergence of functional near-infrared spectroscopy (fNIRS) has opened new avenues for neonatal brain research. This narrative review provides a comprehensive synthesis of recent advancements in fNIRS for neonatal brain monitoring. We aim to delineate the technical principles of fNIRS, critically evaluate its applications in developmental assessment and clinical care, and discuss its future translational potential. By consolidating this evidence, this review clarifies the unique value of fNIRS, addresses the need for a consolidated framework in this rapidly evolving field, and identifies key challenges to guide future research. As a non-invasive, portable, and motion-resilient optical imaging technique, fNIRS is particularly well-suited for bedside assessment and naturalistic observation of brain function. Relying on the principle of neurovascular coupling, fNIRS measures changes in cerebral oxygenation to detect neural activity, thereby providing novel insights into cognitive development, pathological processes, and therapeutic responses in neonates. With continuous advancements in technology and methodology, fNIRS applications in neonatology have expanded markedly, facilitating significant progress in areas such as sensory processing and clinical evaluation of brain injuries. This review provides a comprehensive analysis of recent developments in fNIRS for neonatal brain monitoring. It outlines the technique's foundational principles, technical characteristics, advantages and limitations, and explores its applications in developmental assessment, clinical surveillance, and disease diagnostics, while offering perspectives on its clinical utility and future directions.

## 1 Introduction

The neonatal period represents a pivotal window for brain development, during which functional brain monitoring plays a vital role in the early detection of neurodevelopmental abnormalities and the implementation of targeted intervention strategies. The high plasticity of neonatal brain structures, combined with their distinct metabolic characteristics, makes them especially susceptible to insults such as hypoxia and ischemia (1). Although conventional neuroimaging modalities such as MRI and CT offer high-resolution structural visualization, their application for real-time functional assessment in the neonatal intensive care unit (NICU) is constrained by limitations in portability, exposure risks to ionizing radiation, and practical challenges related to continuous bedside monitoring (2). Against this backdrop, functional near-infrared spectroscopy (fNIRS) has emerged as a critical technique, offering non-invasive, motion-tolerant, and bedside-compatible imaging capabilities for neonatal brain research (3, 4).

**Table 1:** Research progress of fNIRS in neonatal auditory stimulation.

Classification	Researchers	Stimulation/intervention method	Main brain regions activated	Significance
Speech discrimination	Uchida-Ota et al. (30)	Maternal voice vs. stranger's voice	Bilateral frontotemporal network	Maternal voice recognition ability is formed during the fetal period, derived from prenatal auditory experience accumulation
Phoneme Learning	Wu et al. (31)	5 h speech training (natural vowels vs. reversed vowels)	Inferior frontal region, superior temporal region, left inferior parietal lobe	Confirms the neural plasticity mechanism of ultrafast phoneme learning in the neonatal brain
Grammar Perception	Martinez-Alvarez et al. (32)	Grammatical speech vs. ungrammatical speech	Right temporal region	Basic grammar recognition ability exists at birth
Accent Discrimination	Giordano et al. (33)	Native accent vs. foreign accent	Differentiation between left and right hemispheres	Early formation of language division of labor in left and right hemispheres (neural encoding of language social attributes exists at birth)
Native Language Emotional Prosody Perception	Zhang et al. (34)	Native language (emotional prosody such as happiness, anger) vs. foreign language emotional prosody stimulation	Right superior temporal gyrus	Provides evidence for prenatal language experience shaping brain function
Musical Components	Ren et al. (35)	Music with timbre and rhythm changes	Left superior temporal gyrus	The auditory cortex of premature infants has the ability to process multiple musical elements, and maturity is related to corrected gestational age
Pattern Recognition	Nallet et al. (36)	AAB pattern vs. ABC pattern	Bilateral temporal lobes, frontoparietal lobes	Neonates can automatically extract the rule structure of music, and pattern recognition ability may be a precursor to language/cognitive development; musical stimulation enhances frontotemporal-occipital connections and promotes the development of audiovisual pathways

**Table 2:** Applications of fNIRS in neonatal clinical nursing research.

Nursing intervention measures	Core research findings	Clinical application value
Kangaroo Mother Care (KMC)	Activates the frontal, somatosensory, and motor cortices of premature infants	Supports the promotion of KMC in premature infant care to reduce mortality and promote neurodevelopment
Oral glucose	Reduces pain-related cortical activation	Provides simple and effective non-pharmacological analgesia
Maternal body odor	Activates the bilateral olfactory cortices of full-term infants and extremely premature female infants	Soothes emotions and relieves pain responses
Light intervention (Regular Flash Stimulation)	Enhances the connection between the left frontotemporal lobe and visual cortex	Offers a new direction for early visual development intervention to promote neonatal visual function

**Table 3:** Current challenges and future development directions of fNIRS technology.

Challenge area	Problem	Solution direction
Technical level	Spatial resolution is insufficient, and monitoring of deep brain regions is limited	Combine high-resolution probe design, multi-wavelength light source optimization, and mathematical modeling algorithm improvement to enhance signal analysis capability
Standardization issues	Probe positions, data processing flows, and analysis methods vary significantly across studies, leading to low result comparability.	Establish international standard operating procedures for neonatal fNIRS detection
Limitations in clinical research	Most studies are cross-sectional observations, lacking large-sample longitudinal data and long-term follow-ups, with unclear causal relationships	Conduct multi-center, prospective cohort studies to explore the real-time guiding role of fNIRS in neuroprotective interventions
Multimodal integration development direction	Single technology struggles to comprehensively reveal the neural mechanisms of neonatal brain development	Combine fNIRS with EEG, MRI, ultrasound, and other technologies to achieve integrated analysis of structural-functional-metabolic information

fNIRS enables the assessment of regional brain activity by detecting fluctuations in oxygenated hemoglobin (HbO₂) and deoxygenated hemoglobin (HHb) concentrations, in accordance with the neurovascular coupling mechanism (5). Since its pioneering application to neonatal visual cortex monitoring by Meek et al. in 1,998 (6), the scope of fNIRS research has expanded considerably—from localized functional assessments to whole-brain network analyses (7). Today, fNIRS is widely utilized not only to monitor the dynamic maturation of functional brain networks in healthy neonates but also to support early diagnosis and monitoring of conditions such as hypoxic-ischemic encephalopathy (HIE) and to evaluate the efficacy of neurorehabilitation strategies (8).

Given the accelerated trajectory of neonatal brain development and the potentially severe outcomes of brain injury, it is imperative to elucidate the technical foundations, recent advances, and clinical implications of fNIRS in this domain. This review provides a comprehensive examination of fNIRS technology, detailing its principles, strengths, and limitations, while highlighting its applications in developmental assessment, clinical monitoring, and disease diagnosis. Additionally, we explore the clinical relevance of fNIRS and offer insights into its future prospects for translational research and practice. This article is presented as a narrative review, offering a comprehensive and critical overview rather than a systematic appraisal of all available evidence. Our objective is to synthesize the dispersed literature across basic and clinical domains, thereby clarifying the current state of the art and highlighting the pathway for clinical translation of fNIRS in neonatology. In typical neonatal fNIRS studies, sample sizes generally range from 20 to 40 participants, with monitoring durations varying from several minutes to half an hour. The number of channels used commonly ranges between 16 and 64. Experimental designs include both task-based (e.g., auditory stimulation with speech syllables or musical tones, visual stimulation with flickering patterns or face-like images) and resting-state paradigms, often conducted during awake or natural sleep states to accommodate the behavioral characteristics of neonates.

## 2 Literature search strategy

To ensure transparency, a systematic literature search was conducted despite the narrative nature of this review. PubMed, Web of Science, and Google Scholar were searched for publications from 2000 to 2024 using keywords: “fNIRS” OR “functional near-infrared spectroscopy” AND “neonate” OR “newborn” AND (“brain development” OR “clinical monitoring” OR “HIE”). The search focused on original research and high-impact reviews in English, excluding studies on older populations or where fNIRS was not the primary focus. This strategy ensured the identification of relevant literature to provide a comprehensive and critical overview of the field.

## 3 fNIRS overview

### 3.1 Basic principles and technical characteristics

Near-infrared spectroscopy (NIRS) was first introduced by Jöbsis in 1977 (9), who demonstrated that near-infrared light can effectively penetrate biological tissues. This discovery paved the way for its use in monitoring tissue oxygenation. Gagnon first confirmed that near-infrared spectroscopy (NIRS, full name: Near-Infrared Spectroscopy) can identify changes in cerebral oxygenation status in preterm infants when they encounter adverse events, and marked the corresponding reference (10). The fundamental principle of NIRS lies in the distinct optical absorption properties of oxyhemoglobin and deoxyhemoglobin: oxyhemoglobin absorbs more light at shorter wavelengths (∼700 nm), while deoxyhemoglobin exhibits stronger absorption at longer wavelengths (∼800 nm) (7). A typical NIRS system comprises light sources and detectors (11). The sources emit near-infrared light (700–900 nm) into biological tissues, and the detectors capture the attenuated light after it has undergone absorption and scattering. The measured intensity is then used to calculate local concentrations of oxyhemoglobin, deoxyhemoglobin, and oxygen saturation, thereby reflecting tissue oxygen metabolism (5). NIRS was first applied in neuroimaging studies in 1993 (12). When specifically employed for assessing brain functional activity, the method is referred to as functional near-infrared spectroscopy (fNIRS). As a form of multi-channel NIRS monitoring, fNIRS enables simultaneous evaluation of cerebral oxygen metabolism across multiple brain regions. This capability, coupled with topographical mapping, enhances its localization precision (13).

As an optical neuroimaging modality, fNIRS is grounded in the principle of neurovascular coupling. When neurons in a specific brain region become active during cognitive or sensory tasks, localized cerebral blood flow increases, resulting in elevated oxyhemoglobin levels and decreased deoxyhemoglobin levels (14). fNIRS tracks these hemodynamic fluctuations to infer the functional state of the targeted brain region. It is a non-invasive, bedside-compatible imaging tool that is resistant to motion artifacts (15) and impervious to electromagnetic interference, allowing concurrent use with magnetic medical equipment (16). These features render fNIRS particularly advantageous for neonatal brain imaging and make it a valuable component of multimodal neuroimaging approaches in infants.

The efficacy of fNIRS in capturing brain functional activity has been validated through numerous foundational studies. For instance, Hoshi and Tamura reported significant increases in HbO₂ and total hemoglobin (HbT) in task-relevant cortical regions during complex cognitive processing (17). Kato et al. observed rapid changes in HbO₂ and deoxyhemoglobin (HbR) levels in the visual cortex of healthy adults under photic stimulation, followed by a return to baseline (18). Similarly, Villringer et al. identified the expected hemodynamic patterns in the prefrontal and occipital cortices during cognitive and visual tasks, respectively (19). Notably, the high motion tolerance of fNIRS systems has made them indispensable in infant neuroimaging, particularly in studies exploring the neural substrates of early facial recognition (20, 21). In a longitudinal study, Ichikawa et al. demonstrated that infants exhibit earlier neural responses to frontal facial views compared to lateral ones (22). Collectively, these findings underscore the utility of fNIRS in probing critical neural processes in early social cognition and affirm its viability as a method for detecting task-evoked hemodynamic responses, thereby offering robust technical support for infant neuroscience research.

### 3.2 Advantages and limitations of fNIRS

Compared with other techniques for monitoring neonatal brain function—such as functional magnetic resonance imaging (fMRI), amplitude-integrated electroencephalography (aEEG), and positron emission tomography (PET)—functional near-infrared spectroscopy (fNIRS) presents distinct advantages while also exhibiting certain inherent limitations.

One of the key strengths of fNIRS lies in its exceptional portability and suitability for bedside use. The equipment is compact, often wireless, and can be applied directly at the infant's bedside without the need for relocation, making it especially advantageous for continuous monitoring of critically ill neonates in incubators or during routine caregiving activities such as feeding (5, 11). Additionally, fNIRS is non-invasive and safe. It employs near-infrared light (700–900 nm) that poses no ionizing radiation risk (in contrast to CT or PET), and requires only gentle contact with the scalp using flexible optodes and headbands, although care must be taken to ensure the inte grity of fragile neonatal skin is preserved. Importantly, the procedure does not necessitate sedation or strict immobilization (3). From a practical standpoint, fNIRS exhibits strong motion artifact resistance and operational convenience. It can tolerate minor spontaneous movements (e.g., limb activity) (23), features quick setup and measurement durations (often within minutes), and allows for repeated bedside recordings—enabling dynamic tracking of cerebral function over time (24). Technically, fNIRS offers high temporal resolution (typically around 10 Hz, and up to 100 Hz), which is adequate for capturing rapid hemodynamic changes (25). Its penetration efficiency is enhanced in neonates due to their thinner scalp and reduced hair density (26). Moreover, unlike aEEG, fNIRS is highly resistant to electromagnetic interference and can be reliably used in proximity to MRI scanners or in electrically noisy clinical environments (1), making it an ideal component of multimodal neuroimaging strategies.

Nonetheless, fNIRS has several notable limitations. Its spatial resolution (approximately 2–3 cm) and penetration depth (approximately 1–2 cm) are relatively limited. Compared to fMRI (sub-millimeter) and PET (millimeter-level resolution), fNIRS cannot distinguish activity in closely adjacent cortical areas such as gyri and sulci with high precision (5). The limited penetration restricts the assessment to superficial cortical regions, making it unsuitable for evaluating deeper brain structures, including white matter pathways, basal ganglia, hippocampus, cerebellum, and brainstem (3). In addition, fNIRS signals are vulnerable to physiological noise—such as superficial scalp blood flow, cardiac pulsation, and respiratory rhythms—necessitating advanced signal processing algorithms (e.g., short-separation channels, PCA, or ICA) for effective artifact removal. Another constraint is that fNIRS typically yields only relative, rather than absolute, concentrations of HbO₂ and HHb, limiting its ability to generate fully quantitative data. As a result, comparisons between individuals are inherently semi-quantitative and may lack consistency (27). Furthermore, standardization across studies remains a challenge. There is no universally accepted protocol for optode placement on the infant scalp, which can introduce variability in spatial localization and compromise inter-study comparability (4). However, a Polhemu s digitization system can be used to align the emitter detector arrays in the headcap with defined landmarks (fiducials) on the head (Broadmann's areas) (28). Lastly, the signal-to-noise ratio (SNR) in single-subject measurements is relatively low, often requiring group-level averaging to enhance data reliability. This limitation currently hinders the clinical utility of fNIRS in real-time individualized diagnostics, such as the precise localization of epileptogenic foci (5). We have rivised that while fNIRS provides sufficient temporal resolution (typically around 10 Hz) to detect neurovascular coupling, it remains lower than that of electroencephalography (EEG). Similarly, although fNIRS is resistant to electromagnetic interference, its signal quality can be compromised by ambient near-infrared light sources, which may limit its application in outdoor settings or in combination with other near-infrared-based systems such as certain motion capture devices.

## 4 fNIRS applications in neonatal brain functional development assessment

### 4.1 Sensory and cognitive functional development

Functional near-infrared spectroscopy (fNIRS) offers a powerful and non-invasive means to investigate early sensory processing and cognitive development in neonates. Its portability, safety, and tolerance to motion make it ideally suited for capturing the brain's dynamic plasticity during the critical first days and weeks of life, both in natural sleep and wakeful states (29). By measuring task-evoked or resting-state changes in regional hemoglobin concentrations, fNIRS has elucidated the neural foundations of advanced functions in neonates, including auditory perception, language processing, musical cognition, and emotional-social awareness (4). Moreover, fNIRS has expanded its utility to the study of multisensory integration and the developmental trajectory of functional brain networks.

#### 4.1.1 Auditory and language processing

A growing body of evidence indicates that neonates possess surprisingly sophisticated speech discrimination abilities at birth. For example, Uchida-Ota et al. examined 37 neonates aged 2–7 days and observed significantly enhanced functional connectivity within the bilateral frontotemporal network in response to maternal voice stimuli (30). These findings suggest that prenatal auditory exposure may support the early formation of voice recognition circuitry. Further extending this work, Wu et al. demonstrated that neonates are capable of phoneme learning within hours of birth. After just 5 h of exposure to speech training, the hemodynamic response latency to natural vs. reversed vowel stimuli in the inferior frontal cortex significantly decreased. By 7 h, marked increases in neural activation were detected in the superior temporal and left inferior parietal cortices, indicating a remarkably rapid neuroplastic response to linguistic input (31). In terms of syntactic awareness, Martinez-Alvarez et al. reported that neonates exhibit stronger right temporal cortex activation in response to syntactically ungrammatical speech, implying an innate sensitivity to linguistic structure (32). Similarly, Giordano et al. discovered that neonates are capable of discriminating accent features, with left-lateralized activation observed for native accents and right-hemisphere activation for foreign accents. This asymmetrical pattern suggests that the neonatal brain encodes social-linguistic cues immediately after birth (33). Additionally, research by Zhang et al. revealed that neonates are highly sensitive to the emotional prosody of their native language. Activation levels in the right superior temporal gyrus increased significantly in response to emotionally expressive speech, and the degree of activation positively correlated with gestational age (34), offering compelling evidence for the influence of prenatal language exposure on early brain development.

#### 4.1.2 Music processing capacity

Music, as a non-linguistic yet highly structured auditory stimulus, plays a distinct role in shaping early brain development. Ren et al. (35) investigated late preterm infants aged 3–15 days and found significant hemodynamic changes in the left superior temporal gyrus in response to variations in musical timbre, dynamics, and rhythm. These results suggest that even in the earliest days of life, the auditory cortex demonstrates functional maturity in processing multiple dimensions of musical input, with the degree of neural differentiation positively correlated with corrected gestational age. In a separate study, Nallet et al. employed piano tone sequences with differing structures—specifically AAB vs. ABC patterns—and observed more pronounced inverse hemodynamic responses in the bilateral temporal and frontoparietal cortices when neonates were exposed to repetitive AAB patterns (36). These findings indicate that neonates are capable of spontaneously extracting structural rules from auditory sequences, a foundational form of pattern recognition that may underlie later language and cognitive development. Importantly, musical stimulation was also shown to enhance functional connectivity between the frontotemporal and occipital regions, suggesting that music may support the maturation of audiovisual integration pathways through cross-modal neural interactions.

#### 4.1.3 Emotion and social cognition

fNIRS research has significantly advanced our understanding of the neural mechanisms underlying emotion processing in neonates and how these mechanisms evolve during early development. For example, neonates aged 0–4 days are capable of differentiating emotional tones in maternal speech. Compared to neutral prosody, emotionally charged speech—such as expressions of happiness, fear, or anger—elicits increased neural activity in the right superior temporal gyrus. Notably, happy speech induces stronger activation in the left superior frontal gyrus and left angular gyrus, suggesting an early neural bias toward processing positive emotions (34). In a longitudinal comparison, Zhang et al. (37) examined responses to emotional prosody in neonates and 1-year-old infants. They found that neonates exhibited stronger functional connectivity during exposure to happy speech, whereas older infants displayed greater connectivity in response to angry speech. This developmental shift from a “positive bias” to a “negative bias” may reflect adaptive changes in attentional priorities, potentially enhancing the ability of older infants to detect and respond to environmental threats. Additionally, tactile stimulation—particularly through Kangaroo Mother Care (KMC)—has been shown to significantly activate frontal, somatosensory, and motor cortices in preterm infants (38). This early skin-to-skin contact not only supports the integration of multisensory input but also appears to stabilize emotional regulation by strengthening functional connectivity between the prefrontal cortex and limbic system.

### 4.2 Resting-state brain network research

Resting-state functional near-infrared spectroscopy (fNIRS) offers a valuable non-invasive method for investigating spontaneous brain activity and functional connectivity in neonates without the need for task engagement. This approach is particularly effective in evaluating the early development of brain network architecture. The technique operates by measuring inter-regional connectivity strength, analyzing network topological features, and tracking oscillatory dynamics through phase differences in oxygenated and deoxygenated hemoglobin signals—commonly referred to as hemoglobin phase oxygenation and deoxygenation (hPod) (39). Watanabe et al. (40) were among the first to validate the physiological relevance of hPod in infants. Their study involved monitoring term infants, early preterm (23–33 weeks gestational age), and late preterm (34–36 weeks gestational age) neonates during quiet sleep. While no significant differences in hPod were observed at birth, distinct developmental trajectories emerged across groups. Specifically, the rate of change in hPod lagged in preterm infants compared to their term counterparts. These findings suggest that hPod, as detected by fNIRS, serves as a sensitive biomarker for evaluating the maturation of cerebral circulation, metabolic regulation, and neurovascular coupling.

#### 4.2.1 Association between brain networks and neonatal behavioral traits

Resting-state functional connectivity in neonates has been shown to correlate significantly with individual differences in temperament, including traits such as activity level, emotional regulation, and adaptability. Kelsey et al. (41) reported that neonates exhibiting stronger functional connectivity between the frontal cortex and limbic system performed better on behavioral adaptability assessments. These results suggest that early behavioral phenotypes may be rooted in underlying variability in brain network development. In an extension of this work, the same research team incorporated metagenomic sequencing and found that the composition of the gut microbiota also plays a role in modulating brain connectivity. Specifically, a higher relative abundance of Bacteroidetes compared to Firmicutes was associated with stronger frontal lobe connectivity, while overrepresentation of Proteobacteria was linked to reduced connectivity and increased negative affectivity (42). These findings provide empirical support for the existence of a functional “microbiota–gut–brain axis” during the neonatal period. Furthermore, sleep state exerts a notable influence on resting-state brain networks. Using a multimodal fNIRS–EEG approach, Lee et al. (43) discovered that interhemispheric connectivity was enhanced during active sleep, whereas quiet sleep was characterized by predominantly intrahemispheric, localized connectivity. This dynamic switching likely reflects the sleep cycle's modulation of information integration processes in the neonatal brain.

#### 4.2.2 Brain network developmental trajectories

Resting-state fNIRS is highly sensitive to developmental trajectories in brain connectivity that are dependent on gestational age. Arimitsu et al. (44) demonstrated that frontotemporal functional connectivity was significantly stronger in preterm infants born at or beyond 30 weeks of gestation compared to both term infants and those born before 30 weeks. Moreover, infants ≥30 weeks GA exhibited more rapid increases in connectivity following birth. These findings suggest that 30 weeks gestational age represents a critical threshold for accelerated brain network maturation. In contrast, preterm neonates born before 30 weeks gestation possess immature neural architectures and are therefore more susceptible to disruptions in connectivity, particularly due to perinatal complications such as periventricular leukomalacia. This vulnerability increases their risk for later neurodevelopmental disorders. Importantly, functional lateralization begins to emerge early in postnatal life. A longitudinal fNIRS study conducted in infants aged 3–9 months revealed distinct developmental patterns: from 3 to 6 months, local processing efficiency was primarily concentrated in the left temporal cortex, while from 6 to 9 months, global efficiency of the left hemisphere—reflecting integrative information processing—showed a marked increase (1). This progressive hemispheric specialization forms the neural substrate for emerging language and social cognition capabilities. These findings offer compelling neuroimaging evidence to support the implementation of neuroprotective strategies in high-risk preterm populations. By enabling continuous monitoring of brain network dynamics, resting-state fNIRS may facilitate the early identification of infants at neurodevelopmental risk and inform timely interventions—such as non-invasive neuromodulation techniques like transcranial magnetic stimulation—to promote adaptive network remodeling.

## 5 fNIRS applications in clinical monitoring and disease assessment

### 5.1 Pain assessment and care optimization

Functional near-infrared spectroscopy (fNIRS) offers an objective, neuroscientific approach to evaluating pain perception and optimizing neonatal care strategies by capturing real-time changes in cerebral hemodynamics (Tables 1–3). Unlike traditional behavioral pain assessments, which rely on subjective observation and scoring, fNIRS allows for direct measurement of somatosensory cortical activation in response to painful stimuli. These neural responses have been shown to correlate with clinical pain scores, but with greater sensitivity—particularly valuable for preterm infants who are unable to verbally express discomfort and are frequently subjected to invasive procedures (45). In the context of care optimization, fNIRS has demonstrated that Kangaroo Mother Care (KMC)—a skin-to-skin contact method—significantly activates the frontal, somatosensory, and motor cortices in preterm neonates (46, 47). These neurophysiological findings align with World Health Organization recommendations promoting KMC to reduce preterm infant mortality and enhance neurodevelopmental outcomes. Beyond pain detection, fNIRS also provides empirical insight into how sensory environments affect neonatal brain responses. For instance, studies involving olfactory stimulation have shown that preterm infants at 31 weeks corrected gestational age can detect and integrate noxious odors via trigeminal and pain-related neural pathways. Interestingly, administration of oral glucose was found to attenuate such pain-related cortical activation (48). Moreover, maternal body odor was found to selectively activate bilateral olfactory cortices in term neonates and extremely preterm females, highlighting the importance of tailoring sensory interventions to gestational age and sex (49). Additional research has demonstrated that rhythmic light stimulation can enhance cross-modal connectivity between the left frontotemporal region and the visual cortex, suggesting a potential role for early visual training interventions in supporting multisensory development.

### 5.2 Brain injury and neurodevelopmental risk

Leveraging its unique capability for dynamic monitoring and excellent potential for multimodal integration, functional near-infrared spectroscopy (fNIRS) demonstrates significant clinical value in the assessment of neonatal brain injury and the prediction of neurodevelopmental outcomes. This technique is particularly well-suited for high-risk neonates with immature cerebral oxygen metabolism regulation, offering a powerful tool for early risk identification and guiding timely intervention strategies.

#### 5.2.1 Cerebral oxygenation monitoring in preterm infants

Preterm infants are particularly vulnerable to disturbances in cerebral oxygenation due to immature cerebral autoregulatory mechanisms, which impair their ability to maintain stable oxygen supply relative to metabolic demand. This imbalance places them at heightened risk for both hypoxic and hyperperfusive injury. Functional near-infrared spectroscopy (fNIRS) allows for continuous, non-invasive monitoring of regional cerebral tissue oxygen saturation (rSO_2_), making it possible to detect dynamic changes in perfusion patterns in real time. Numerous studies have demonstrated that abnormal fluctuations in rSO_2_—such as frequent or extreme desaturations and overshoots—are significantly associated with the development of brain injuries in preterm infants, particularly intraventricular hemorrhage and white matter damage (50). When used in conjunction with transcranial Doppler ultrasound to measure cerebral vascular resistance indices, fNIRS provides a more comprehensive evaluation of cerebral autoregulation. This multimodal approach enhances the accuracy of clinical assessment and supports more informed decision-making in the management of high-risk neonates (5).

#### 5.2.2 Neonatal asphyxia and hypoxic-ischemic encephalopathy (HIE) assessment

Hypoxic-ischemic encephalopathy (HIE) is a devastating form of neonatal brain injury resulting from impaired cerebral blood flow and oxygen delivery during the perinatal period. It remains a leading cause of long-term neurological sequelae—including epilepsy, cerebral palsy, and motor or cognitive impairments—as well as neonatal mortality (51). Current diagnostic and prognostic approaches for HIE rely on a combination of clinical history, Apgar scores, blood gas analysis (e.g., degree of acidosis), and neuroimaging findings (52). However, the window for effective neuroprotective intervention is narrow, making early and accurate assessment essential for improving outcomes and reducing disability. fNIRS provides new opportunities in this domain by enabling real-time monitoring of cerebral oxygenation and hemodynamic changes. In one study, researchers evaluated infants with HIE by measuring changes in HbO_2_, HHb, and total hemoglobin (tHb) in the prefrontal cortex before and after music stimulation. They found that the degree of change in tHb was negatively correlated with clinical severity, suggesting fNIRS can serve as an early biomarker for both disease severity and therapeutic response (53). Supporting this, Hou et al. (54) demonstrated significantly lower rSO_2_ levels in infants with HIE compared to healthy controls. Following oxygen therapy, affected infants exhibited increased HbO_2_ and rSO_2_, alongside decreased HHb levels, visually confirming treatment efficacy. Ye et al. (55) further expanded fNIRS applications by showing that it can effectively monitor cerebral oxygenation in neonates experiencing intrauterine distress, offering real-time feedback on the success of interventions during labor and delivery. Together, these findings highlight the unique clinical value of fNIRS in the context of HIE. It allows for continuous, non-invasive monitoring of cerebral oxygen metabolism and perfusion, providing crucial objective data for early diagnosis, severity assessment, and personalized treatment planning in neonates at risk of hypoxic-ischemic injury.

#### 5.2.3 Early biomarkers for neurodevelopmental disorders

fNIRS holds significant promise for the early detection of neurodevelopmental disorders by identifying objective, brain-based biomarkers that precede the onset of clinical symptoms. By analyzing functional brain network topologies and activation patterns, fNIRS enables early screening for conditions such as autism spectrum disorder (ASD), attention deficit hyperactivity disorder (ADHD), and cerebral palsy (CP). It is crucial to note that disorders like ASD and ADHD cannot be definitively diagnosed in the neonatal period based on current criteria, as their core symptoms unfold over later development. Therefore, the early neurobiological markers identified by fNIRS typically require longitudinal tracking into infancy and beyond to accurately distinguish true risk and understand the emerging disease trajectory. Abnormalities detected in the neonatal or early infancy period may serve as early warning indicators of future developmental challenges.

In the context of ASD, resting-state fNIRS has revealed atypical network features, including reduced global efficiency, altered small-world architecture, and diminished long-range connectivity within the right frontal cortex. More notably, the efficiency of the deoxygenated hemoglobin (HbR) network appears to decline with age in ASD infants—opposite to the typical developmental trend (56). Machine learning algorithms trained on fNIRS-derived connectivity metrics (e.g., medial prefrontal cortex synchrony) have achieved early ASD classification accuracies as high as 83.3% (57). In social cognition tasks, infants with ASD risk exhibit reduced HbO₂ responses in the left frontotemporal region, reflecting impaired activation in brain areas critical for social processing (58). These early deviations support fNIRS as a valuable tool for both understanding ASD pathophysiology and implementing neonatal risk stratification.

For ADHD, early-stage abnormalities such as decreased global efficiency in the prefrontal–parietal network and disruptions in small-world properties have also been identified through resting-state fNIRS. Importantly, fNIRS-guided neurofeedback training—where real-time activation in the right prefrontal cortex is monitored and adjusted—has shown therapeutic benefits (59). Machine learning models have exceeded 80% diagnostic accuracy, partly due to distinct patterns such as reduced right prefrontal activation during Stroop tasks in ADHD (but not ASD) infants, offering a possible objective basis for differential diagnosis (60). In addition, deficits in processing angry facial expressions have been linked to atypical right prefrontal function. Improvements in right prefrontal activation following pharmacological treatment have been found to correlate with symptom remission, indicating the potential of fNIRS as a monitoring tool throughout early intervention (59).

In the case of CP, particularly in neonates at high risk due to perinatal brain insults (e.g., prematurity, asphyxia, or hemorrhage), fNIRS's portability and motion robustness make it ideal for early neurofunctional assessment (61). Abnormal motor cortex activation patterns and disrupted interhemispheric coordination have been observed during finger movement tasks in infants who later develop hemiplegia (62). Research by De Campos et al. further revealed that in children with CP, the lesioned hemisphere exhibits paradoxically heightened activation during bimanual tasks—often exceeding that of the non-lesioned hemisphere (63). These atypical activation signatures may emerge before overt motor symptoms appear, supporting fNIRS as a powerful early tool for evaluating neural reorganization and identifying infants at elevated risk for CP.

Most of the existing studies are exploratory in nature and limited by single center small sample sizes. In the future, multi center studies should be conducted as much as possible and sample sizes should be expanded to better evaluate the clinical relevance and reproducibility of observation results.

### 5.3 Application of fNIRS in monitoring brain function in hypoxic newborns undergoing therapeutic hypothermia

fNIRS can serve as a tool for monitoring brain function in hypoxic infants undergoing therapeutic hypothermia. A study by Wintermark et al. (64) confirmed that the cerebral perfusion indicators measured by fNIRS have a significant correlation with MRI results, providing key evidence for the reliability of fNIRS in real-time and non-invasive assessment of cerebral perfusion status during hypothermia treatment.

## 6 Challenges and future directions

Although functional near-infrared spectroscopy (fNIRS) has shown considerable promise for neonatal brain function monitoring, its widespread clinical implementation still faces multiple challenges. At the technical level, the limited spatial resolution and insufficient penetration depth for monitoring deep brain structures remain significant obstacles. Addressing these issues will require advancements in high-resolution optode design, the optimization of multi-wavelength light sources, and the refinement of signal processing through more sophisticated mathematical modeling algorithms. Standardization represents another critical hurdle. Substantial variability in probe placement, data acquisition procedures, and analytical methodologies across studies leads to poor reproducibility and low inter-study comparability. The establishment of internationally recognized standard operating procedures (SOPs) for neonatal fNIRS is urgently needed to ensure consistency and reliability across research and clinical settings. In clinical research, most current studies remain cross-sectional in nature, with a paucity of large-sample, longitudinal datasets. This limitation hinders the exploration of causal relationships between fNIRS-derived metrics and long-term neurodevelopmental outcomes. To bridge this gap, future efforts should focus on multi-center, prospective cohort studies aimed at validating the real-time utility of fNIRS in guiding neuroprotective interventions. Moreover, the integration of multimodal technologies presents a promising direction. Combining fNIRS with other neuroimaging modalities such as electroencephalography (EEG), magnetic resonance imaging (MRI), and cranial ultrasound could enable a more comprehensive analysis of neonatal brain structure, function, and metabolism. This multimodal approach may offer deeper insights into the complex neural mechanisms underlying early brain development. Another critical challenge in neonatal fNIRS studies is data quality and attrition (i.e., data exclusion). Owing to factors such as infant movement and state instability, data attrition rates in some studies can reach 20%–30%, which must be carefully considered in both research design and clinical translation.

### 6.1 Limitations and research gaps

The current evidence base for neonatal fNIRS applications has several important limitations. Most studies are characterized by small sample sizes and single-center designs, which limit the generalizability of findings. There is also a notable scarcity of longitudinal studies that track neurodevelopmental outcomes, hindering the validation of fNIRS as a predictive biomarker. Furthermore, research on the use of fNIRS for monitoring interventional efficacy and its application in resource-limited settings remains underdeveloped.

### 6.2 Priorities for future research

To address these gaps, future work should focus on: (1) conducting large-scale, multicenter longitudinal studies; (2) establishing standardized, age-specific protocols; and (3) exploring the role of fNIRS in guiding and evaluating neuroprotective interventions. Addressing these priorities will be crucial for translating technical promise into clinical impact.

## 7 Conclusions and prospects

Functional near-infrared spectroscopy (fNIRS) has emerged as a critical modality for neonatal brain function assessment, owing to its non-invasiveness, portability, and relative robustness against motion artifacts. It provides unique and real-time insights into early neurodevelopmental processes. In basic neuroscience research, fNIRS has enhanced our understanding of the maturation of sensory processing, the formation of resting-state networks, and the dynamics of neurovascular coupling during infancy. From a clinical standpoint, fNIRS contributes to translational medicine by serving as a valuable biomarker platform for the early detection of brain injury in high-risk neonates, screening for neurodevelopmental disorders, and informing individualized intervention strategies. These applications are made possible through the objective quantification of cerebral hemodynamics related to pain and stress responses, abnormal oxygen metabolism, and disrupted network topology. Despite its promise, current limitations include restricted depth penetration—particularly its inability to visualize the deep brain nuclei such as the basal ganglia and thalamus—as well as the absence of standardized protocols across institutions. Future research should prioritize the integration of multimodal imaging, advanced machine learning techniques, and large-scale longitudinal cohorts. These efforts will further unlock the potential of fNIRS in neonatal neurocritical care, enabling dynamic, continuous monitoring of developmental trajectories and supporting a closed-loop system for brain risk identification and targeted neuroprotection.
